# Impact of Patient Navigation on Timeliness of Diagnosis and Treatment, and Adherence to Treatment in Middle Income Asian Country

**DOI:** 10.1200/GO.22.29000

**Published:** 2022-05-05

**Authors:** Maheswari Jaganathan, Yamuna Rajoo, Nadia Rajaram, Azuddin Mohd Khairy, Nur Hidayati Zainal, Jasiah Zakaria, Mazwela Adi, Shahtirah Halim, Fizati Sabtu, Siti Zubaidah Sharif, Nur Shuhadah Jaafar, Farhana Harzila Mohd Bahar, Nik Azim Nik Abdullah, Adibah Ali, Sharifah Ashrina, Rokayah Julaihi, Isabella Menon, Kavitha Muniandy, Hani Zainal, Mallika Muniandi, France Olovia Roimin, Aini Fatimah Ghazali, Clara Chong Ching Ling, Norlia Rahim, Nurul Ain Tajuddin, Fazilah Ambia, Nurizzati Cheng, Norija Sapiee, Soo-Hwang Teo, Mohamed Yusof Abdul Wahab

**Affiliations:** ^1^ Cancer Research Malaysia, Subang Jaya, Malaysia; ^2^ Pink Ribbon Centre, Hospital Tengku Ampuan Rahimah, Klang, Malaysia; ^3^ Pink Ribbon Centre, Hospital Tunku Jaafar, Seremban, Malaysia; ^4^ Pink Ribbon Centre, Hospital Queen Elizabeth II, Kota Kinabalu, Malaysia; ^5^ Pink Ribbon Centre, Hospital Umum Sarawak, Kuching, Malaysia

## Abstract

Women in low to middle-income countries (LMICs), such as in Malaysia, face complex barriers to healthcare because of social, economic, geographic, and other interrelated factors, which can limit their access to timely, affordable, and effective breast cancer care. Patient Navigation has emerged as a cost-effective and impactful approach to enable women to access care.

**METHODS:**

We have established a hospital-based partnership model between a non-profit organization and national healthcare service to address the barriers faced by low-income women to improve the timeliness of diagnosis and treatment and reduce poor adherence to treatment. The navigation team provides resources required to overcome financial and logistic barriers, counseling to address knowledge and emotional needs and to ensure treatment adherence.

**RESULTS:**

To determine the effectiveness of the program, timeliness of diagnosis and treatment, and rates of treatment adherence were compared with a historical cohort from the same hospital. The proportion of patients who met the healthcare system's targets for timeliness to cancer diagnosis increased from 50.4% to 67.3% (*P* < .05) and that for initiation of primary treatment increased from 36.5% to 49.5% (*P* < .05). The overall treatment default rates reduced from 8.6% to 2.6% (*P* < .05).

**CONCLUSION:**

In summary, Patient Navigation is feasible and potentially effective for addressing barriers to cancer care and could be a key element of cancer control in LMICs.

